# Diversity of endohelminth parasites of fish from Sali-Dulce River tributary (Tucumán province, Northwestern Argentina)

**DOI:** 10.1007/s11686-026-01395-x

**Published:** 2026-09-16

**Authors:** Lorena G. Ailán-Choke, Fabiana Cancino, Geraldine Ramallo

**Affiliations:** 1 Centro de Ecología Aplicada del Litoral, Consejo Nacional de Investigaciones Científicas y Técnicas, Ruta Provincial 5, Km 2,5, Corrientes, CP. 3400 Argentina; 2 Setor de Parasitologia, Departamento de Biodiversidade e Bioestatística, Instituto de Biociências, Universidade Estadual Paulista-UNESP, Distrito de Rubião Júnior, Botucatu, 18618-689 São Paulo Brasil; 3 Instituto de Vertebrados, Fundación Miguel Lillo, Tucumán, Argentina; 4 Instituto de Invertebrados, Fundación Miguel Lillo, Tucumán, Argentina

**Keywords:** Endoparasites, Endorheic basin, Neotropical Region, Nematoda, Siluriformes, Characiformes

## Abstract

**Purpose:**

Medina-Salas River is a tributary of Sali-Dulce endorheic system, one of the most important basins of Northwester Argentina. Despite, many endemic fish species can be found in this endorheic system, and its ichthyological diversity is affected by anthropogenic activities, the diversity of endohelminth parasite of fish in this river remains unexplored. This study aimed to analyse the structure and composition of endohelminth communities of fish species from lower sector Medina-Salas River.

**Methods:**

Fish belonging to seven species (*Bryconamericus iheringii*, *Jenynsia tucumana*, *J. lineata*, *Trichomycterus corduvensis*, *T. alterus*, *T. spegazzinii*, and *Heptapterus qenqo*) were examined for endohelminth parasites. Ecological analyses were performed at the component community and infracommunity levels.

**Results:**

A total of 1269 endoparasites belonging to six taxa (5 Nematoda and 1 Cestoda) were found in the intestine or abdominal cavity from a total of 951 fishes screened. *Contracaecum* sp. was the only taxon found in the larval stage. The helminth taxon that displayed the highest prevalence, mean intensity and mean abundance was the nematode *Cucullanus pinnai* of *H. qenqo*. Most taxa of parasitic helminth exhibit the typical aggregated pattern of distribution within host populations. All component communities presented a low richness and diversity values. *Jenynsia tucumana* showed the most diverse helminth component community.

**Conclusion:**

This study is the first comprehensive ecological survey on the diversity of endohelminth fauna of freshwater fish species from Northwest Argentina, presenting six new host records contributing to the knowledge of the real patterns of helminth parasites diversity from Neotropical fish.

## Introduction

Parasites probably represent the most successful and widely distributed form of life; however, they are the most overlooked in management and conservation of biological and ecosystem resources [1–3]. These organisms can exert strong selective pressures on their host populations, affecting their morphology, fecundity, reproduction, behaviour, and survival [4, 5]. Indeed, parasite populations and communities could be used as indicators of environmental stress, food web structure, and biodiversity [6]. High parasite diversity in aquatic environments has been associated with a healthy ecosystem [6–8]. Several studies have analysed the patterns of endohelminth diversity in wild freshwater fish across different ecosystems in the Neotropics [5, 8–12]. However, freshwater fish populations from Argentina have received little attention [13–18], and no studies have been performed in fish hosts from Medina-Salas River, a tributary of Sali-Dulce endorheic system.

The Sali-Dulce River endorheic basin is the largest in Argentina, extending from northwestern to central Argentina. This system presents two sectors, divided by Rio Hondo dam, located between the provinces of Tucumán and Santiago del Estero [19]. This basin harbours approximately 93 fish species, including both native and introduced taxa. Its ichthyofauna is mainly of Paranoplatense origin [20]. In the upper basin, within Tucumán Province, about 66 native fish species have been recorded, many of which are of economic, recreational, and sanitary importance [19]. This sector is strongly affected by agro-industrial activities, urbanization, and the presence of dams that disrupt fish migratory routes [19].

The Medina-Salas River, a typical mountain stream, is a tributary of this basin, located in its upper section within the province of Tucumán. This river presents a torrential hydrological regime, with intense rainfall during summer and dry periods in winter [21–23]. This is a predominantly rural area, with scarce population density, where agriculture, livestock production [21], and aggregate extraction are the main activities (pers. obs.). Despite the knowledge about the diversity of fish in Sali-Dulce basin, no surveys have specifically addressed the ichthyofauna of the Medina–Salas River. Given its location within the upper Salí–Dulce basin, its fish assemblage is expected to be broadly consistent with that reported for the Sali-Dulce system.

Prior to the present study, Ramallo [24] was the first to record and describe a new species of *Rhabdochona* in *Bryconamericus iheringii* (Boulenger,1887) and reporting *Rhabdochona acuminata* (Molin, 1860) in *Jenynsia lineata* (Jenyns, 1842). However, ecological studies were not performed to date. Therefore, this study aims to characterise the structure and composition of endohelminth communities of seven species of fish from the lower Medina-Salas River, a tributary of Sali-Dulce basin.

## Materials and Methods

### Sampling of Fish Hosts and Parasitological Procedures

**Fig. 1:**
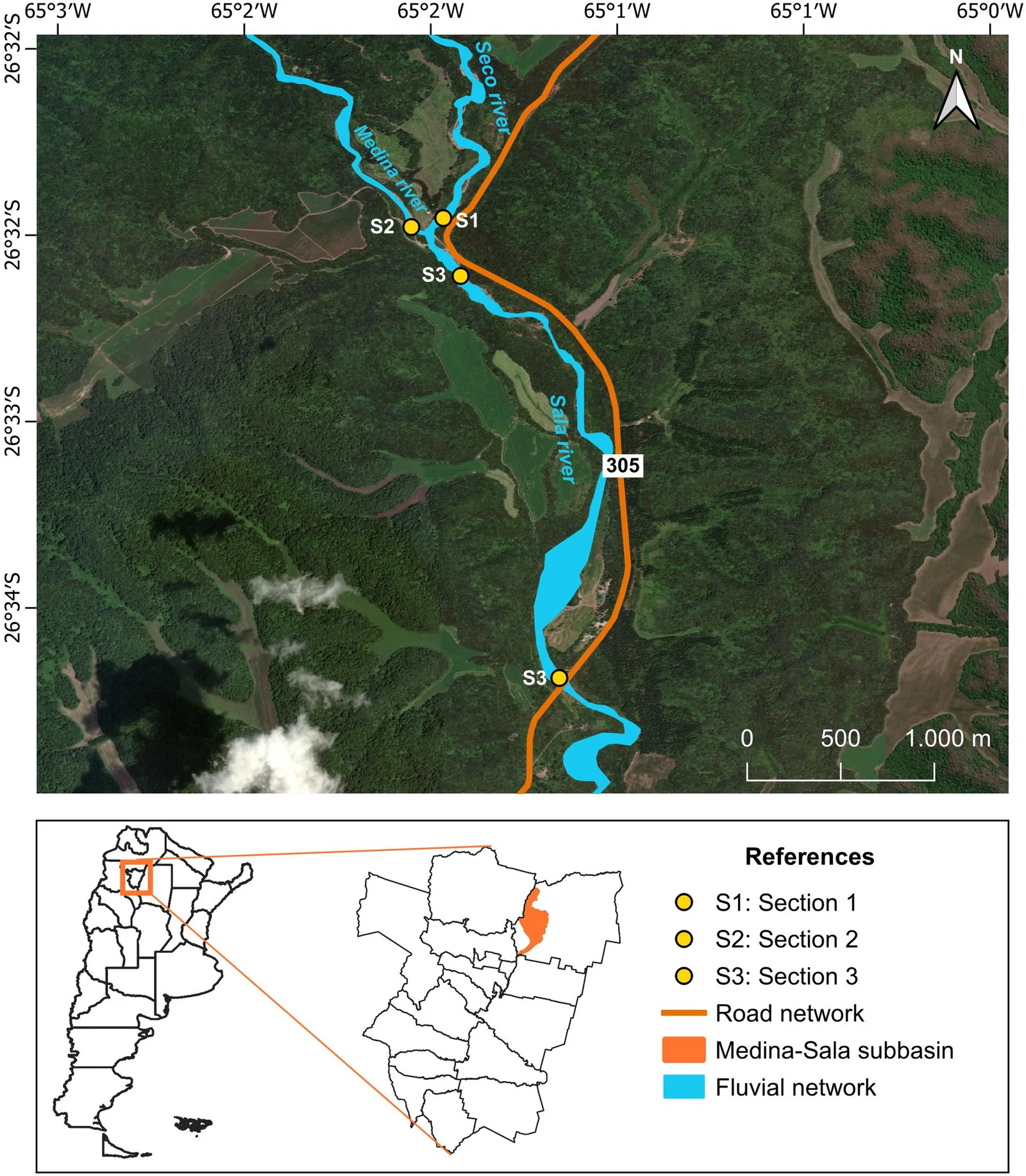
Map of the study area

The samplings were conducted in different points of Medina-Salas River: 1º section (26º32’20.10” S – 65º01’44.57” O), 2º section (26º32’26.15” S – 65º1’48.41” O) and 3º Sect.  (26°32’25.2” S; 65°01’47.3” O; 26º33’52.70” S – 65º01’24.60” O). All sampling sections are located at an approximate altitude of 800 m a. s. l. and within the same river basin, with a maximum distance of approximately 3 km between sampling points (Fig. 1). The sampling sites represent similar microhabitats and were therefore considered broadly comparable in terms of environmental conditions.

Fish belonging to seven species were collected between May 2019 and March 2021 using hand nets, drag nets, and electrofishing equipment (Table 1). The collected fish were fixed in 10% formalin and then preserved in 70% ethanol, and some specimens were fixed and preserved in 96° ethanol until examination. Each host was weighted (g), measured for standard length (mm) and examined for endohelminth parasites. Their taxonomic identification, nomenclature and classification were updated according to Froese & Pauly [25].

**Table 1:** Number of fish hosts collected for sampling periods in Medina-Salas River, endorheic basin of the Salí-Dulce River, Burruyacú Department, Tucumán province, Argentina.

Host species	May 2019	July 2019	October 2019	March 2021	TNH
Trichomycteridae					
*Trichomycterus alterus*	32	48	46	21	147
*Trichomycterus spegazzinii*	0	20	6	6	32
*Trichomycterus corduvensis*	29	5	6	5	45
Heptapteridae					
*Heptapterus qenqo*	14	30	27	5	76
Anablepiidae					
*Jenynsia tucumana*	101	150	190	36	477
*Jenynsia lineata*	2	14	8	0	24
Stevardiidae					
*Bryconamericus iheringii*	36	69	36	9	150

Total number of specimens collected for host species (TNH)

The corporal cavity and all organs were examined under a stereomicroscope. Helminths were examined under a Leica DMLB light microscope for taxonomical identification. Nematodes were cleared with lactophenol prior to examination. Cestodes were examined based on available morphological characters; however, due to their poor preservation condition, they could not be adequately processed for staining. The taxonomic identification of each endohelminth taxa followed specific literature, pertinent to each taxon [24, 26–28]. Voucher specimens were deposited in the Colección de Invertebrados (Sección Helmintos y Anélidos) (CH-N-FML; CH-C-FML), Fundación Miguel Lillo, San Miguel de Tucumán, Argentina.

### Statistical Analysis

Prevalence, mean intensity, and mean abundance of the helminth parasites were calculated following Bush et al. [29]. Discrepancy index (D), described by Poulin [30] was used to evaluate the spatial distribution of parasites based on their abundance in the host population [30]. This index ranges from 0 to 1, interpreted as: D = 0, all hosts harbouring a similar number of parasites; D = 1, all parasites found in a single host. This analysis was calculated using the Quantitative Parasitology 3.0 software [31].

To determine the structure of helminth communities of each host population, analyses were performed at component community level (all helminth species in a host population of each species) and at infracommunity level (all helminth species found in an individual host of each species) [29, 32]. The following indices of the parasite community were calculated: (a) parasite species richness, that is the number of species in an assemblage, which was estimated at component community and infracommunity levels; (b) Simpson index (1 − D) to analyze the diversity of parasites, which was calculated at component community level ranging from 0 (low diversity) to 1 (high diversity); (c) Shannon-Wiener diversity index, that was estimated at component community level, this index measures the order or disorder in a system, by attributing greater weight to rare species, and was relatively independent of sample size [33, 34]; (d) Berger-Parker index was used to demonstrate the dominance of helminth species, which was calculated at the component community level [32]. Shannon-Wiener, Simpson and Berger-Parker indices were calculated using the software PAST (version 4.3) [35].

Species accumulation curves were estimated for the component community of each host species to evaluate sampling completeness by assessing the relationship between parasite species richness and sampling effort [36]. Curves were generated in R using the specaccum function of the vegan package with the random method.

## Results

Seven species of host fish were examined (Table 2). Of the 951 host specimens examined for endohelminths, 223 (Percentage of parasitized hosts = 23.5%) were infected with one or more taxa of parasites. A total of 1269 endoparasites were collected mostly from the intestine or the abdominal cavity of the fish. Adult endohelminths represented 99.4% (1261/1269) of all collected specimens, while larval forms accounted for only 0.6% (8/1269). Table 3 summarizes the infection parameters for each host–parasite association, including the number of helminth specimens, prevalence, mean intensity, mean abundance, infection site, and discrepancy index (D).

**Table 2:** Body parameters of fish of Medina-Salas River, endorheic basin of the Salí–Dulce River, Burruyacú Department, Tucumán province, Argentina

Host species	Number of fish examined			Weight (g)	Standard length (mm)
	Male	Female	Juvenile		
*Trichomycterus alterus*	31	95	21	1.2 ± 0.8	39.2 ± 7.7
*Trichomycterus spegazzinii*	17	11	4	3.4 ± 3.6	53.9 ± 14.6
*Trichomycterus corduvensis*	14	12	19	4.0 ± 3.4	58.3 ± 17.8
*Heptapterus qenqo*	53	18	5	18.9 ± 14.6	125.9 ± 39.6
*Jenynsia tucumana*	191	286	0	1.3 ± 0.5	34.3 ± 1.3
*Jenynsia lineata*	8	16	0	1.0 ± 0.8	34.9 ± 7.8
*Bryconamericus iheringii*	69	79	2	4.0 ± 1.7	54.8 ± 7.9

**Table 3:** Total number of parasites (TNP); Prevalence (P%); mean intensity (MI); mean abundance (MA); index of Discrepancy (ID) and infection site (IS) of the helminths found in the seven fish species from Medina-Salas River, endorheic basin of the Salí–Dulce River, Burruyacú Department, Tucumán Province, Argentina.

Host species	Parasite taxa	TNP	*P*(%)	MI ± SD	MA ± SD	ID	IS
*Heptapterus qenqo*	Cestoda						
	Eucestoda gen. sp*(CH-C-FML #07940)	3	3.9	1.00 ± 0	0.04 ± 0.20	0.95	Intestine
	Nematoda						
	*Cucullanus pinnai* * (CH-N-FML #07932)	522	86.8	7.90 ± 6.00	6.90 ± 6.20	0.48	Intestine
*Trichomycterus alterus*	Nematoda						
	Rhabdochona acuminata* (CH-N-FML #07949)	26	13.6	1.30 ± 0.47	0.48 ± 0.18	0.88	Intestine
*Trichomycterus spegazzinii*	Nematoda						
	*Spirocamallanus huacraensis* (CH-N-FML #07948)	2	6.2	1.00 ± 0	0.06 ± 0.25	0.91	Intestine
	*Rhabdochona acuminata* (CH-N-FML 07950)	14	18.8	2.33 ± 1.69	0.44 ± 1.34	0.87	Intestine
*Trichomycterus corduvensis*	Nematoda						
	*Spirocamallanus huacraensis* (CH-N-FML #07935)	2	4.40	1.00 ± 0	0.04 ± 0.21	0.94	Intestine
	*Rhabdochona acuminata* * (CH-N-FML #07947)	4	6.70	1.33 ± 0.58	0.09 ± 0.36	0.92	Intestine
*Jenynsia tucumana*	Nematoda						
	*Contracaecum* sp. (larvae) * (CH-N-FML # 07945)	7	1.30	1.17 ± 0.41	0.01 ± 0.14	0.91	Corporal cavity
	*Rhabdochona acuminata* * (CH-N-FML #07946)	12	2.30	1.09 ± 0.30	0.03 ± 0.17	0.97	Intestine
*Jenynsia lineata*	Nematoda						
	*Contracaecum* sp. (larvae) (CH-N-FML #07933)	1	4.20	1.00 ± 0	0.04 ± 0.20	0.92	Corporal cavity
	*Rhabdochona acuminata* (CH-N-FML #07934)	6	4.2	6.00 ± 0	0.25 ± 1.22	0.92	Intestine
*Bryconamericus iheringii*	Nematoda						
	*Rhabdochona fabianae* (CH-N-FML #07931)	670	71.10	6.32 ± 6.15	4.50 ± 5.90	0.629	Intestine

*Species recorded for the first time in this host species

MI and MA are showed as mean ± standard deviation

Six endohelminth parasite taxa were found: five nematodes (four adults and one larvae) and one cestode. Of the adult helminth taxa found, only the nematode *R. acuminata* and *Spirocamallanus huacraensis* Ramallo, 2008 occurred in more than one host species, more specifically five and two host species, respectively (Table 3). The larval nematode *Contracaecum* sp. also occurred in two host species (Table 3).

The most abundant taxon was *Rhabdochona fabianae* Ramallo, 2005 from *B. iheringii* (*n* = 670) followed by *Cucullanus pinnai* Travassos, Artigas & Pereira, 1928 from *Heptapterus qenqo* Aguilera, Mirande & Azpelicueta, 2011 (*n* = 522); whereas the larval nematode *Contracaecum* sp. from *J. lineata* showed the lowest abundance (*n* = 1), followed by *S. huacraensis* from *Trichomycterus corduvensis* Weyenbergh, 1877 and *T. spegazzinii* (Berg,1897) (*n* = 2 in each host species), and Eucestoda gen sp. from *H. qenqo* (*n* = 3). The helminth taxon showing the highest value of prevalence (P) (86.8%), mean intensity of infection (MI) (7.9 ± 6.0) and mean abundance (MA) (6.9 ± 6.2) was the nematode *C. pinnai* from *H. qenqo*, followed by *R. fabianae* from *B. iheringii* (Table 3). Most taxa of parasitic helminths exhibit the typical aggregated pattern of distribution within host populations, with values of D (Discrepancy index) close to 1; except for *C. pinnai* from *H. qenqo* and *R. fabianae* from *B. iheringii* (see Table 3 for details).

**Fig. 2:**
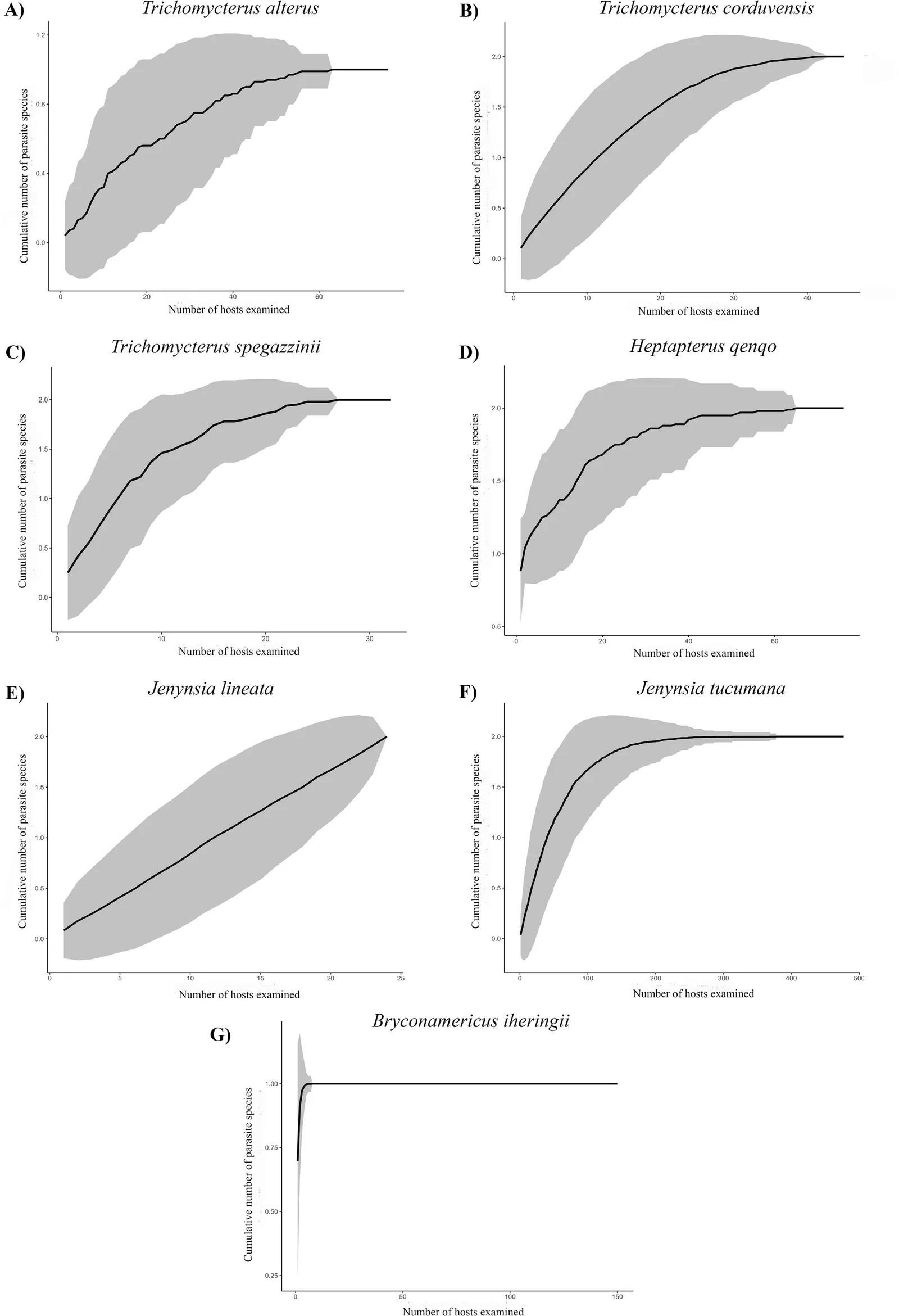
Species accumulation curves of endohelminth parasites for each of the seven fish host species examined in the Medina-Salas River, an endorheic basin of the Salí–Dulce River, Burruyacú Department, Tucumán Province, Argentina. Shaded areas represent ± 1 standard deviation around the mean species richness.** A** *Trichomycterus alterus*,** B** *Trichomycterus corduvensis*, **C** *Trichomycterus spegazzinii*, **D** *Heptapterus qenqo*, **E** *Jenynsia lineata*, **F** *Jenynsia tucumana*, and **G** *Bryconamericus iheringii*

In general, the helminth component communities of all host species examined presented a low richness and diversity (Table 4). The helminth communities of *Jenynsia tucumana* Aguilera & Mirande, 2005 and *J. lineata* were the only ones harbouring endohelminth larvae; whereas the remaining helminth communities presented only adult helminth taxa. The helminth component community of *J. tucumana* was the most diverse followed by that from *T. corduvensis*, according to the diversity index values (Table 4). However, the helminth component communities of *B. iheringii* and *Trichomycterus alterus* (Marini, Nichols & La Monte, 1933), were represented by only one species, with the lowest diversity indices values. At the infracommunity level, the communities were represented by low richness and diversity, ranging 0–2 for each infracommunity, with a high proportion of fish infected with only one endohelminth species (Table 4). Species accumulation curves reached an asymptote for six of the seven host species, indicating that the sampling effort was sufficient to characterize their component communities (Fig. 2). In contrast, the curve for *J. lineata* did not reach an asymptote (Fig. 2E).

**Table 4:** Descriptors and diversity indices of endohelminth communities from seven host species collected in the Medina-Salas River, endorheic basin of the Salí-Dulce River, Burruyacú Department, Tucumán Province, Argentina.

Host species	*P* (%)	NT	TNP	*R*	MR	SWI	SI	BPI
*Heptaterus qenqo*	86.84	2	525	0–2	0.82 ± 0.48	0.04	0.01	0.99
*Trichomycterus alterus*	13.61	1	26	0–1	0.14 ± 0.34	0	0	1
*Trichomycterus spegazzinii*	21.88	2	16	0–2	0.25 ± 0.45	0.38	0.22	0.88
*Trichomycterus corduvensis*	11.11	2	6	0–1	0.10 ± 0.32	0.64	0.45	0.67
*Jenynsia tucumana*	3.56	2	19	0–1	0.04 ± 0.19	0.66	0.47	0.63
*Jenynsia lineata*	8.33	2	7	0–1	0.08 ± 0.29	0.41	0.25	0.86
*Bryconamericus iheringii*	70.67	1	670	0–1	0.71 ± 0.46	0	0	1

Percentage of parasitized hosts (P%), total number of taxa (NT), total number of parasites (TNP), Range of species richness (R), and the Simpson (SI), Shannon-Wiener (SWI), and Berger- Parker (BPI) indices were calculated at the component community level. Mean species richness (MR) was calculated at the infracommunity level

The Berger-Parker values for each component community indicated that generally the component communities were strongly dominated by one single species (BPI = 0,9). Although the component communities from *J. tucumana* and *T. corduvensis* were weakly dominated by a single species, their Berger–Parker index values (= 0.6) indicate a more even distribution of abundances compared to other communities (BPI = 0.9), where a single species strongly dominate (Table 4). All component communities were dominated by nematode species.

## Discussion

The study of endohelminths infecting seven fish species from a tributary of the Sali-Dulce River (Argentina) identified six parasite taxa, with nematodes being the most frequent and diverse taxa. These results agree with previous studies about the endohelminth fauna of freshwater fish from neotropical rivers [8, 18, 37]. From the six taxa identified, only the nematode, *Contracaecum* sp. was found in the larval stage; hence, adult helminths were predominant.

Considering that the different taxa of endohelminth present a complex life cycle and require two or more hosts to complete their development, they can use invertebrates as intermediate hosts and fish as secondary, paratenic or definitive hosts [8]. Thus, the composition of endohelminth communities of fish, namely if it consists of adult (using it as definitive host) or larval helminths (using it as intermediate or paratenic host), can provide data about the position of the animal within trophic network and its diet composition [38]. In our study, the finding of larvae of *Contracaecum* sp. only in *J. lineata* and *J. tucumana* with low infection (prevalences of 4.2% and 1.3%, respectively) seem to indicate that these hosts could be used as paratenic hosts, that may be prey for piscivorous birds, where the adult stages of this helminth taxa could be found [8, 38–41]. *Jenynsia lineata* (= *Jenynsia multidentata*) and *J. tucumana* are preferentially generalist omnivorous, that mainly predates on both plants (e.g., algae and seagrass) and animals (e.g., microcrustaceans: copepods, amphipods; aquatic insect larvae: dipterans, ephemeropterans; polychaetes) [42, 43]. Thus, microcrustaceans could act as the first intermediate host of these larvae of *Contracaecum* sp. Moreover, Montes & Martorelli [17] also observed larvae of *Contracaecum* sp. parasitizing specimens of *J. multidentata* collected from the Salado Relief Channel on Samborombón Bay and the Sauce Chico River near the city of Bahia Blanca (Argentina). However, they reported a great number and high dominance of parasitic larval stages (not only *Contraecum* sp., but also different morphotypes of metacercariae), indicating that this fish could be used as the food item for others vertebrate species, different to that observed in the present study with predominance of adult helminths both in *Jenynsia* spp. and in other host species.

The remaining host species display endoparasite communities composed only of helminths in the adult stage. The differences in the proportion of larval and adult helminths observed in the present study, could be explained not only by diet, but also by habitat use, which can drive the position of these fish in the food webs of this environment [11, 44, 45]. Available evidence for the genus *Trichomycterus* suggests that the group is represented by generalized benthonic predators of invertebrates [46]. *Trichomycterus corduvensis*, is a generalized predator of aquatic benthonic insects, with an occasional appearance of vegetal elements in its diet; however, the feeding patterns of *T. alterus* and *T. spegazzinii* are unknown [46, 47]. Likewise, *B. iheringii* is a small carnivorous fish that feeds larvae of Trichoptera, Diptera, and Ephemeroptera, and secondarily on chrysophyte and chlorophyte algae. It inhabits still, vegetated waters with benthopelagic habits [25, 48, 49]. The generalist diet and benthopelagic habitat use of *Trichomycterus* spp. and *B. iheringii* might expose them to a broader variety of item-prey that could act as intermediate host of nematode parasites found in each host population.

The life cycle of *Rhabdochona* spp. is little known, but the hitherto data indicate that the main intermediate hosts of these helminths are mayflies (Ephemeroptera) and less often some other aquatic insects such as caddisflies (Trichoptera) or stoneflies (Plecoptera) [50]. Thus, our findings suggest that infection by *R. acuminata* and *R. fabianae* in *Trichomycterus* spp. and *Jenynsia* spp. and *B. iheringii*, respectively, occurs via ingestion of Ephemeroptera larvae, which likely harbour the larval stages of these parasites. In the case of *S. huacraensis* that parasitizes *T. spegazzinii* and *T. corduvensis* with low infection (prevalence of 6.2% and 4.4%, respectively), its life cycle is unknown, but studies on *Spirocamallanus* species have shown that generally different species of copepods may act as intermediate hosts of camallanids [8, 51]. In the present study, *Trichomycterus* spp. could acquire the infection through feeding on copepods containing the infective stages of *S. huacraensis*.

Despite the lack of diet data for *H. qenqo*, some species of *Heptapterus* have been reported as invertivorous, feeding on a large proportion of benthonic invertebrates [52]. The high infection by *C. pinnai* in *H. qenqo* (prevalence of 86.8%) is likely to occur through the ingestion of larvae invertebrates (Odonata, Ephemeroptera, and Megaloptera nymphs), which may contain the larval stages of this cucullanid. However, Køie [53] reported that *Cucullanus cirratus* Müller, 1777 may have a life-cycle that involves copepod as transport hosts, and a fish as intermediate host (gobies or cod fry). Postcyclic development may occur in gadoids when an infected cod is consumed by another cod (cannibalism). Similar findings were reported by Tanzola et al. [54], who hypothesized that *C. pinnai* presents a heteroxenic pattern of transmission involving *R. quelen* as definitive host and *B. iheringii* (Characidae) as the intermediate host. Thus, an integrative approach using morphological and molecular analyses would be necessary to identify the host species involved in the heteroxenous life cycle of *C. pinnai*, recorded in the present study. These approaches would allow us to assess whether insect larvae act as intermediate or paratenic hosts, or whether another host, maybe a fish, is involved in the life cycle.

Cestoda was the least represented endohelminth group, in terms of species richness, comprising only one taxon found. *Heptapterus qenqo* was the unique host infected by this parasite, with a low infection (prevalence of 3.9%). The low prevalence observed may be related to the complex heteroxenous life cycle of this parasite, which depends on the availability of suitable intermediate hosts and successful transmission to the definitive host [55]. Regarding digeneans, no records were found in the examined fish species. Although freshwater gastropods, which may act as first intermediate hosts for digeneans, have been recorded in the study area [56], the establishment and development of digenean life cycles depend on a set of biotic (presence of suitable intermediate and definitive hosts) and abiotic (light, temperature, and circadian rhythms, which regulate cercarial emergence) factors. Therefore, the reasons for the absence of digeneans in the present study remain uncertain and further studies addressing the occurrence of potential intermediate hosts and other ecological factors are needed to better understand the dynamics of transmission of these parasites in the study area. However, previous studies in other Argentinian freshwater systems have reported digeneans belonging to families Lecithasteridae, Derogenidae and Allocreadiidae parasitizing *J. lineata*, *J. alternimaculata* (Anablepidae), *Hatcheria macraei* (Girard, 1855) (Trichomycteridae) and *H. qenqo* (Heptapteridae), indicating that both phylogenetically related fish species and some of the same host species examined in the present study can serve as hosts for these parasites in other freshwater systems [57–60].

All endohelminth component communities showed low species richness and low to moderate diversity. Similar results were observed at the infracommunity level for each host species (Table 4). Similar patterns have been reported in other neotropical freshwater systems, with low species richness and diversity recorded at both the infracommunity and component community levels across different fish species [61–63]. However, other studies conducted in neotropical fish species from different hydrographic systems, have reported richer and more diverse endohelminth communities, suggesting that parasite community structure may vary considerably among freshwater ecosystems as a consequence of ecological differences among host–parasite systems [11, 15, 64, 65], The lower richness and diversity values observed in the present study may reflect differences in the host ecology (e.g. diet and habitat use), environmental conditions associated with this mountain stream, and the local availability of intermediate hosts, all of which may directly influence the transmission dynamics and establishment of helminth parasites [8, 62, 66, 67].

*Jenynsia tucumana* had the most diverse helminth community of this study; whereas *B. iheringii* and *T. alterus* presented the lowest (Table 4). Although parasite richness was similar among helminth communities from different host species (Table 4), differences in diversity indices may suggest variation in evenness. Diversity indices are sensitive to both species richness and the relative abundance of each species within a community [36]; thus, diversity increases with richness and reaches a maximum when all species are equally represented, i.e., when the value of evenness is maximum [68]. In contrast, the other host species showed higher dominance patterns in their endohelminth communities, possibly reflecting more specialized host–parasite interactions, like the component community from *B. iheringii* composed by only one species, *R. fabianae*. This nematode seems to be specific to this host, because it has not been recorded in other fish species [69]. In addition, differences in recruitment rates of individuals to the community can result in one or a few numerically dominant species and many rare ones (with low abundance) [70]. The prevalence of infection in the intermediate hosts of helminths that serve as prey for fish, and the rates at which fish consume different prey types could determine the recruitment rates [70].

To date, only one study has been published on the endohelminth community of one host species analyzed in the present study. Montes & Martorelli [17] studied the helminth fauna of *J. lineata* (= *J. multidentata*) from two different environments and reported a rich-species community, composed by 16 taxa, with the digenean metacercariae as the most diverse and dominant taxa. In contrast, the component community of *J. lineata* was composed of only two endohelminth species. This difference may reflect geographical and ecological variations (environmental conditions and intermediate/definitive host availability) among the study areas. Although species accumulation curves indicated that the sampling effort was sufficient to characterize the helminth component communities of the remaining host species, the curve for *J. lineata* did not reach an asymptote, indicating that its parasite richness may have been underestimated due to limited sampling effort. Therefore, comparisons between the two studies should be interpreted with caution. Whereas Montes & Martorelli [17] found metacercariae of *Ascocotyle* (*Phagicola*) *diminuta* Stunkard & Haviland (1924) and larvae of *Contracaecum* sp. to be the most prevalent taxa; the adult nematode *R. acuminata* exhibited the highest prevalence in the present study. Regarding other host species, the available data are related to helminth species description or host/geographical records [24, 26, 57, 60, 69,  71].

It is worthy to note that the component communities from *Jenynsia* spp. and *Trichomycterus* spp. share one generalist species, *R. acuminata*, and even the communities of *Jenynsia* spp. and those of *T. corduvensis* and *T. spegazzinii* show the same composition within each genus (Table 2). These patterns could be related to the host biological features, namely phylogenetically close species that share diet, use habits and are sympatric may show similarities in their helminth fauna, since host switching among parasite species is facilitated over evolutionary time [72–74].

In conclusion, we present the first comprehensive ecological survey on the diversity of endohelminths of freshwater fish species from Northwest Argentina. The endohelminth fauna was composed of few species, some of which were shared among the fish species analysed, with a high dominance of nematodes. Endohelminths were found in larval and adult stages, indicating that some fish species may act as intermediate, paratenic and definitive hosts. This work contributes to the knowledge about the composition and structure of endohelminth communities, as well as infection parameters, in the host species examined at both the component community and infracommunity levels. In addition, six new host records were reported here for the first time. Considering that the studied environment is exposed to different anthropogenic disturbances (agriculture and aggregate extraction), the new data provide a baseline for further studies assessing the effects of human activities on parasite community structure and composition, and their potential use as indicators of environmental changes.

## Data Availability

All data analysed in this study is available in the manuscript.
